# 2020 Grand challenge revisited: removing silos, advancing research to improve overall health

**DOI:** 10.3389/fdmed.2024.1430828

**Published:** 2024-07-11

**Authors:** Martha J. Somerman, Wendy E. Mouradian, Sukirth M. Ganesan

**Affiliations:** ^1^ Frontiers in Dental Medicine, Chevy Chase, MD, United States; ^2^ University of Washington School of Dentistry, Seattle, WA, United States; ^3^ Washington and the Santa Fe Group, New York, NY, United States; ^4^ Department of Periodontics, The University of Iowa College of Dentistry and Dental Clinics, Iowa City, IA, United States

**Keywords:** integrating medicine and dentistry, systemic-oral health research, collaborations across disciplines, oral-systemic association, dental medical integration

## Abstract

This perspective provides strong evidence that the aims set forth when Frontiers in Dental Medicine (FDMED) was launched in 2020, to advance the integration of dental, oral, and craniofacial research with mainstream biomedical practice, underscored the value of interprofessional research collaborations, encouraging studies, publications, and commentaries in this area ripe for continued innovation. The momentum gained toward these aims must continue to inform the public, healthcare providers, researchers, educators, and policymakers so that they can apply the knowledge gained to improve the overall health needs of all our communities.

## Introduction

This perspective is written as a reflection on the Grand Challenge set forth in 2020 for the newly launched Frontiers in Dental Medicine, quote: “The newly launched (June 1, 2020) Frontiers in Dental Medicine (FDMED) has a bold mission and vision focused on ensuring that dental. oral, and craniofacial health and diseases are understood in the context of whole-body health……FDMED will accomplish this by attracting rigorous, innovative investigations, and commentaries from researchers where dental-oral health is an integral part of investigations into underlying health mechanisms to include biological, behavioral, and social factors, clinical approaches, tools and technologies and healthcare delivery models/policies” (1). FDMED remains committed to this mission.

Fortunately, as the field chief editor for FDMED, MS has been fortunate to work with an incredibly insightful and committed Frontiers staff, specialty chief editors, associate editors, special topic editors providing timely Research Topics (*over 140),* and reviewers, resulting in high-quality publications. Many of these publications, with increased publications in disciplines beyond dentistry, emphasize the epidemiologic correlation and mechanistic aspects of oral health and disease states and how these findings advance our understanding of tissues/cells in other regions of the body and inform clinical practice. Moreover, publications from other journals and other venues, such as seminars, conferences, webinars, National Academy of Science, Engineering and Medicine (NASEM) forum, educational programs [inter professional educational (IPE) curricula], practice-based networks, and clinical practice have emphasized the need for transdisciplinary collaborations to delineate the mechanisms for multifactorial diseases from basic to translational to clinical trials to implementation science (2–14).

The attention given to oral-systemic integration is rewarding and yet it is important to reflect on our mission and vision to ensure that we enjoy success going forward. The recent editorial in JAMA, 2023, by M.S. Reddy et al (15) calls for the need for continued attention to oral health research. They pointed out that the 2023 US Preventive Service Task Force (USPSTF) reported that the evidence was insufficient to determine the benefits and harms for oral health conditions and for providing preventive interventions by primary care clinicians for children aged 5–17 years and adults (16–18). The USPSTF report highlights that not only is more research needed but we must communicate the significant evidence to date indicating that good oral health is associated with improved systemic health. Outlined below are some successes to date and directions to consider toward increasing collaborative efforts across disciplines and of equal importance for encouraging the sharing of data, materials, tools, and technologies across disciplines. Some suggested action items to keep this momentum going are provided in Table 1.

**Table 1:** Strategic initiatives for integrating oral health and systemic health.

Focus areas	Action item	Description
**Advancing Transdisciplinary Research from bench to community**	1. Launch an Annual Conference and/or Grand Rounds focused on oral-systemic research and clinical practice	• Organize annual conferences or grand rounds with themes such as Cardiometabolic and Alzheimer's (similar to Gordon conferences) • Include panels with a broad range of healthcare providers and patients to gain a deeper perspective
	2. Identify and communicate science advances affecting clinical practice	• Consider developing a monthly newsletter spearheaded by the Office of Data-Driven Solutions at NIDCR • Establish metrics to evaluate the newsletter's impact
	3. Promote an international committee of students to develop strategies for comprehensive healthcare integration	• Form a committee led by academic organizations to create educational models for comprehensive healthcare • Select representatives from student organizations
	4. Establish a functional open-access Dental, Oral, and Craniofacial (DOC) data repository	• Provide updates on the status of the Office of Data-Driven Solutions portal for data sharing • Share access information through key healthcare organizations such as ADEA, AMA, IADR, ANA, AACN
**Advancing Educational Experiences Across Disciplines**	5. Publish data on interprofessional educational curricula and establish IPE curricula for use by all professional schools	• Expand interprofessional educational programs for student involvement in curriculum development and CE/CME/CNE courses
	6. Establish an international integrated student network to discuss educational models and promote IPE	• Collaborate with global organizations similar to ADEA, AAMC, AACN for sharing educational models and promoting interprofessional education
**Advancing Health Care Models**	7. Promote educational and healthcare systems policy	• Form a team to assist the community in developing and implementing educational and healthcare policies
	8. Develop integrated dental clearance and management forms	• Work with ADA/AMA/ANA leadership to implement standardized referral forms for all healthcare systems
	9. Increase involvement of DOC exams in clinical trials	• Advocate to include DOC exams in clinical trials through IADR's congressional connections
	10. Optimize use of Electronic Health Records (EHR) for integrated medical-dental communication	• Model after schools that have integrated EPIC (and similar EHR) into dental school systems and advocate for its implementation in all dental schools

NIDCR, National Institute of Dental and Craniofacial Research; ADEA, American Dental Education Association; AMA, American Medical Association; ANA, American Nurses Association; AACN, American Association of College of Nursing; CE, Continuing Education; CME, Continuing Medical Education; CNE, Continuing Nursing Education; AAMC, Association of American Medical Colleges; DOC, Dental, Oral, and Craniofacial; HER, Electronic Health Records. The focus areas are ordered for specific reasons:
1) **Advancing Transdisciplinary Research from bench to community:** This focus area lays the foundation for integrating oral health with systemic health by fostering collaboration across disciplines, disseminating research findings, and engaging stakeholders from bench (research) to community (clinical practice). Starting with this focus area demonstrates a commitment to building a solid evidence base and promoting awareness of the interconnectedness of oral and systemic health. 2) **Advancing Educational Experiences Across Disciplines:** Once research initiatives are underway, it's crucial to educate and train professionals across various disciplines about the significance of oral-systemic health integration. By prioritizing educational experiences, you can ensure that future healthcare providers are equipped with the knowledge and skills needed to address oral health within the broader context of overall health. 3) **Advancing Health Care Models:** With a solid research foundation and a well-educated workforce, the next step is to implement integrated healthcare models that incorporate oral health into routine healthcare practices. This focus area addresses policy changes, clinical protocols, and infrastructure enhancements necessary to support seamless collaboration between dental and medical professionals.

### Accelerating multidisciplinary dental-oral-craniofacial research

Our first eBook for FDMED, “Integrating Oral and Systemic Health: Innovations Transdisciplinary Science, Health Care and Policy” (19), emphasized the value of incorporating research focused on the dental-oral-craniofacial (DOC) complex with research focused on other regions of the body. In particular, several authors noted that dental issues (in rodent models and in humans) may be the first signs of disease/disorders affecting overall health, while several articles focused on infections of the oral cavity, e.g., periodontitis, which increase the risk of other diseases, emphasizing the need for transdisciplinary research and healthcare teams for preventing, diagnosing and treating diseases/disorders and the need for integrative models to be part of our healthcare educational programs. At the time we underscored areas where bidirectional, transdisciplinary research, including clinical trials/data analyses have paid off at the clinical level. These included the human microbiome, periodontitis-diabetes, salivary diagnostics, craniofacial conditions, and SARS-CoV-2 pandemic research (20).

Since the launching of FDMED and the vision/goals set forth in 2020, it is gratifying to learn that substantial progress has been made in fostering medical-dental collaborations in the areas mentioned above, from research to education to clinical practice to policy documents. For example, collaborations across disciplines have resulted in the recognition that salivary glands and specific cell types are a locus for infection e.g., SARS-CoV-2 (21, 22) and that salivary fluids are viable assets for diagnosing diseases across the body (23, 24). We refer the reader to several other reviews/editorials/commentaries since 2020 demonstrating increased understanding, because of multidisciplinary approaches, and the benefits of these strategies toward improving healthcare for all communities (2, 15, 25–32). Notably, while recognizing limitations with meta-analysis, Botelho and colleagues concluded that twenty-eight non-communicable diseases (NCDs) were strongly associated with oral diseases including five types of cancer, diabetes mellitus, cardiovascular diseases, depression, neurogenerative conditions, rheumatic diseases, inflammatory bowel disease, gastric helicobacter pylori, obesity, and asthma. These reviews demonstrate advances in defining oral-systemic interactions in health and disease, while bringing attention to the need for further investigations to ensure research findings move from the bench to clinical practice and improve educational and healthcare system policies.

Below is an excellent example where fostering interprofessional engagement, in part attributed to a special funding mechanism at NIH to jump start this area of research (33), has advanced our knowledge and understanding of oral-systemic interactions and informed healthcare delivery systems across medicine and dentistry. This instructive case reminds us of the importance of gathering evidence-based data to confirm research findings and to enable the extension of these findings to clinical settings.

#### The microbiome across systems

The Human Microbiome Project was launched by NIH in 2007 (34) with the goal of mapping the human microbiome, choosing five sites: mouth, nasal, skin, gastrointestinal and urogenital tract. At the time, there was active discussion as to whether this initiative would prove to be of much value toward the NIH's mission of improving the health of all communities. Now, seventeen years later, all communities from researchers to clinicians to patients have gained from this initiative. The data generated by transdisciplinary teams has resulted in an exponential growth of our understanding of the importance of the microbial environment within our body to host-microbial interactions in health and states of disease and increased the awareness of the effects of the oral microbiome on other organs of the body and vice versa, such as the GI tract, cardiovascular system, lungs, and the brain (35–40).

Critically, the distinct characteristics of the oral microbiome, including the compositional differences when compared to other microbial niches such as the gut and skin, have been increasingly understood. For example, the patterns of relative abundances of two common bacterial colonizers, *Streptococcus* and *Bacteroidetes,* are reversed in the oral and gut environments. Defining common and disparate responses of the host to microbiomes, depending on the region of the body, has resulted in a better appreciation of host-microbial-immune interactions (41, 42). Examples of bacteria receiving much attention related to oral-gut interactions are: (a) *Fusobacterium nucleatum*, which in the oral cavity is associated with severe periodontal disease but is linked to colon cancer in the GI tract (43)*;* and (b) *Porphyromonas gingivalis*, a bacterium associated with severe periodontal disease, and overall microbiome dysbiosis (44–48). Several of these articles provided models to explain the oral-gut connection. One such model by S. Kitamoto and his colleagues (40) provides a logical explanation for the ability of pathobionts from the oral cavity to increase, directly and indirectly, the inflammatory condition of the gut.

Beyond oral-gut dynamics, research is actively delving into the oral-lung axis. Following the initial detection of COVID-19 viral presence in saliva, investigations have focused on community translocation through coughing and swallowing, or induced inflammatory responses. Notably, several oral microbiota species like *Capnocytophaga*, *Neisseria*, and *Veillonella* are found in lung fluid of COVID-19 patients (49). And not a surprise, Ehrenzeller et al's systematic review (39), provides evidence that daily tooth brushing is associated with lower rates of hospital-acquired pneumonia. Further, there is mounting evidence that non-ventilator pneumonia is preventable/considerably reduced with implementation of an oral hygiene program (50, 51). Furthermore, the oral-brain axis is under study, revealing potential implications for Alzheimer's disease pathogenesis. Proof of *P. gingivalis* infection compromising blood-brain barrier integrity in mice has demonstrated the role of oral bacteria beyond the oral cavity (52).

And another area where advances have been made is related to HPV oral/pharyngeal vaccines for the prevention of HPV-associated cancer and the need for all healthcare providers to discuss this topic with their patients, and to consider the oral healthcare professional's role as vaccinators (5, 53).

The good news is that oral health and diseases are experiencing greater visibility and recognition from diverse audiences and yet, it is disappointing to observe areas where limited or no progress has been made.

Apologies to the many outstanding researchers not included here, related to oral-systemic interactions. Your voices are needed. We encourage you to submit manuscripts that highlight crosstalk between the oral cavity and other organs.

#### There is always room for improvement

As mentioned above, the USPSTF's report should serve as a reminder to our community and beyond that it is not just evidence-based research data showing strong correlations between oral-systemic health and disease that is needed. We must communicate our findings to diverse audiences.

One example presented below, brings to the forefront the need for more attention to oral-systemic integration in relationship to therapies for treating diseases/disorders from basic science to clinical trials, to educational programs. The focus here is on mineralized tissue diseases/disorders. Importantly, the oral cavity is the only place in one's body where four mineralized tissues, enamel, dentin, cementum, and bone are located together. Moreover, developmentally, teeth have unique properties, selective to each species, allowing for further understanding of genes/proteins regulating the formation of enamel, cementum, and dentin and the consequences of mutations in genes associated with these tissues. Logically, including the DOC (dental, oral, and craniofacial) region in animal models, *in vitro* models, and clinical trials is vital to defining factors influencing the status of hard tissues over a life span and approaches for treating diseases/disorders of hard tissues. Yet too often DOC tissues are not considered in these investigations for their deleterious effects.

#### Emerging medications to treat a variety of disorders: general comment

There has been an exponential growth in new therapies and medications to treat complex diseases and disorders and with this, an increase in *in vivo* and *in vitro models* and clinical trials to evaluate the mechanism of action and safety of these medications. However, too often the effects on DOC cells/tissues/organs are neglected. While clinical researchers will rationalize that there would be added costs, we would argue that inclusion of oral tissue examinations in many clinical trials will save the costs of recognizing the negative and positive oral outcomes of a specific medication after the close of the trial. Of course, this does not mean that all medications being evaluated need to include an oral arm but where a medication logically, based on mechanism of action, may affect oral tissues, such as therapies being proposed to treat hard tissue-associated diseases/disorders, they need to be a part of pre-clinical investigations and clinical trials.

#### Medications used to treat disorders of mineralized tissues

It has been many years since the recognition that bisphosphonates (BPs), used to treat osteoporosis and bone metastatic cancers, may result in osteonecrosis of the jaw in a limited number of individuals (54)*.* When cases of osteonecrosis of the jaw were first reported in cancer patients receiving BPs, drug companies argued it was most likely related to the other medications patients were receiving. However, with intensive investigations, among transdisciplinary researchers and clinicians, it became evident that BPs were the culprit and further, that high doses and long-term therapy coupled with poor oral health increased the risk for BP-mediated osteonecrosis of the jaw (55). Retrospectively, it's easy to realize oral exams should have been part of the clinical trials, given the well-known fact that teeth are very sensitive to alterations in local levels of phosphate/pyrophosphate (56).

Fast forward, newer drugs, targeted to cells associated with bone homeostasis have been developed for treating osteoporosis and metastatic cancers, such as denosumab, a RANKL inhibitor, and teriparatide, a parathyroid (PTH) analog (57). However, once again clinical trials, to the best of our knowledge, have failed to include examination of oral tissues. While these therapies are welcomed additions to improve bone health, side effects have emerged to include the rebound effect (rapid bone loss) after discontinuation of denosumab, when alternative therapies are not provided upon termination (58). And, there have been case studies indicating a similar pattern associated with teeth, i.e., denosumab-mediated external resorption of tooth roots (59, 60). Proposed mechanisms for rebound osteoclast activation are not completely understood but current evidence suggests hyperosteoclast activity with discontinuation of denosumab. Toward this end Somerman and Thumbigere-Math responded to the NEJM review on postmenopausal osteoporosis by Walker and Shane (57) pointing out that oral–dental side effects, which often are inadequately evaluated during medical examinations, could be minimized by the implementation of dental examinations and procedures before antiresorptive treatment is started. To address this issue, they recommended integrating dental clearance and dental management as standard-care practices for patients receiving treatment for osteoporosis. Unfortunately, the authors did not embrace our recommendation (61). The need for future clinical studies to include oral exams and the need for improved communication among all healthcare providers are obvious.

Yet another example we previously highlighted is associated with the rare metabolic disorder, hypophosphatasia (HPP), characterized by low tissue-non-specific alkaline phosphatase (TNAP) activity due to loss of function of *ALPL*, the gene that encodes TNAP. Individuals with mutations in *ALPL* exhibit a broad range of phenotypes including rickets, osteomalacia and dental diseases from perinatal HPP, to infantile HPP, to childhood HPP, to adult HPP, to dental-specific, odonto-HPP (62). Despite the recognition of the dental phenotype and the inclusion of dental analysis in the many animal models of HPP, when clinical trials were performed, using enzyme replacement strategies, oral exams were not included. Now, new gene delivery approaches are in the process of moving forward to clinical trials and yet, as far as we are aware, oral exams are not being included.

Several chronic diseases, notably Type 2 Diabetes (T2D), exhibit a profound association with poor oral health, as evidenced by an elevated prevalence and severity of periodontal disease. The well-established bidirectional relationship between T2D and periodontal disease is further underscored by emerging evidence linking these conditions with obesity and Metabolic Syndrome (MetS) (63–67). For instance, patients with uncontrolled T2D and obesity often present with severe oral diseases, leading to increased treatment costs. Recently, Singh et al. evaluated the periodontal treatment costs of over 3,400 adults classified as normal weight, overweight, and obese. After controlling for covariates and disease severity, patients who were obese had 27% higher periodontal treatment costs than patients who were normal weight. More interestingly, the additional periodontal treatment costs attributable to obesity were more significant than those attributable to either diabetes or smoking (68). In addition, medications to treat these conditions lead to xerostomia and associated complications. Recognizing bidirectional links, the World Health Organization (WHO) advocates for a global health strategy integrating oral health with primary healthcare, emphasizing preventive approaches that both enhance systemic health and curtail dental treatment costs and oral health burden.

Patients with T2D and obesity require interdisciplinary care involving dietetics, nutrition, podiatry, ophthalmology, and psychology. Dentistry, too, must be incorporated into such comprehensive care, surpassing the conventional need for dental clearance before surgery. It is imperative that oral healthcare providers take an active role in informing their medical colleagues about the significance of these oral-systemic connections. Beyond treatment and clinical care, a pivotal insurance policy change, encompassing preventive prophylactic and maintenance appointments for patients with systemic diseases to be implemented in all insurance healthcare plans, is indispensable for delivering comprehensive care. The evidence for including preventive dental care (PDC) in all comprehensive healthcare plans for individuals with diabetes is supported by Lamster et al. (31), who report improved general health when PDC is provided for patients with diabetes. In addition, work by Borah et al. demonstrated that the PDC in patients with diabetes and coronary artery disease (CAD) who were enrolled in expanded dental insurance coverage was strongly associated with significant savings for these chronic disease cohorts. The extended dental program in this study was a medical-dental integrated program that provided additional dental benefits at no cost to the patient and did not apply to the patient's calendar year maximum. Additionally, the deductible and co-insurances were waived, and two additional preventive appointments (cleaning or periodontal maintenance) were paid at 100% and did not apply to the annual maximum allowable benefits. The economic burden studies and cost savings studies from PDC in high-risk chronic disease cohorts emphasize the crucial need for dental coverage to be included in the health plan benefits package to improve overall health and lower costs for enrollees with chronic diseases (31, 68).

## Conclusion: next steps to promote oral-systemic integration

Transdisciplinary research has increased over the past decades with the recognition of the need for diverse strategies and expertise for tackling many unknowns linked to health and disease. Such approaches, some cited in this perspective, have enhanced our knowledge base and in many cases moved knowledge into educational models, policy changes, and clinical practice. However, omission of oral tissues from transdisciplinary investigations leads to their the absence of healthcare delivery systems and healthcare policies in the long term (69).

As stated in our 2020 Grand Challenge, we need to continue to promote communications/networking across disciplines. At that time, we called attention to the Santa Fe Group: https://santafegroup.org; and a resource library for the integration of primary care and oral health, https://resourcelibrary.hsdm.harvard.edu/, which continues to provide excellent resources for activities and progress in the across healthcare systems. Other groups focused in this area include the American Academy for Oral Systemic Health (AAOSH), Care Quest Institute for Oral Health, Marshfield Clinical Research Institute, and very recently, the Office of Data-Driven Solutions at NIDCR/NIH announced that they are building a portal for data sharing across studies. There is a need for cross-disciplinary groups to continue to work together to ensure oral health and disease are considered in the context of total body health. Some areas requiring attention are highlighted in Table 1.

## Data Availability

The original contributions presented in the study are included in the article/Supplementary Material, further inquiries can be directed to the corresponding author.
